# Developing a co-production strategy to facilitate the adoption and implementation of evidence-based colorectal cancer screening interventions for rural health systems: a pilot study

**DOI:** 10.1186/s43058-022-00375-2

**Published:** 2022-12-13

**Authors:** Jungyoon Kim, Paul Estabrooks, Alisha Aggarwal, Analisa McMillan, Khalid Alshehri

**Affiliations:** 1 Department of Health Services Research and Administration, University of Nebraska Medical Center, College of Public Health, 984350 Nebraska Medical Center, Omaha, NE 98168 USA; 2 Department of Health & Kinesiology, College of Health, University of Utah, Salt Lake City, USA; 3 Janssen Pharmaceutical Companies of Johnson and Johnson, Horsham, USA; 4 College of Public Health, University of Nebraska Medical Center, Omaha, USA

**Keywords:** Bundled implementation strategies, Online blueprint implementation strategy, Academic-clinical partnership, Plan-do-study-act cycle, Colorectal cancer screening, Rural primary care, Mixed-method

## Abstract

**Background:**

Evidence-based colorectal cancer screening (CRCS) interventions have not been broadly adopted in rural primary care settings. Co-production of implementation strategies through a bundled approach may be promising in closing this gap by helping rural healthcare practitioners select and implement the best fitting CRCS interventions to the local context. This paper describes the process and outcomes of co-development and delivery of the bundled implementation strategy to improve adoption and implementation of CRCS interventions with two rural clinics.

**Methods:**

We used a bundle of implementation strategies with a core focus on academic-clinical partnership development (strategy 1) and Plan-Do-Study-Act cycles (strategy 2) to identify clinical partner interests/preferences on delivery methods and content needed to facilitate intervention identification and implementation that improves CRCS. We also developed an implementation blueprint for each clinic (strategy 3) through an online blueprinting process based on adapted “Putting Public Health Evidence in Action” (PPHEA) training curriculum. Clinic physicians and staff (*n* = 7) were asked to evaluate the bundled approach based on overall reactions and perceptions of innovation characteristics using 5-point Likert scale. After completing the bundled approach, we collected implementation outcomes and limited intervention effectiveness of the CRCS evidence-based interventions (EBIs) developed through the process.

**Results:**

Our co-production strategy yielded a prototype online blueprinting process consisting of 8 distance-learning PPHEA modules that guide selection and implementation of EBIs tailored to CRCS. Modules were delivered to clinic participants with minor adaptations, using PDSA cycle to improve quality of module contents and formats. Overall, participants in both clinics reported positive reactions toward the bundled approach. Both clinics reported improvements in how they perceived the characteristics of the innovation (the bundled approach) to tailor selected CRCS EBIs. As a result of the bundled strategies, each clinic selected and adopted specific EBI(s) with the varying degrees of implementation and CRCS outcomes.

**Conclusions:**

The bundle of implementation strategies used were feasible and acceptable in rural primary care practices to facilitate the use of EBIs to improve CRCS.

**Supplementary Information:**

The online version contains supplementary material available at 10.1186/s43058-022-00375-2.

Contributions to the literature

- Few studies report on the co-production of implementation strategies for evidence-based colorectal cancer screening (CRCS) in rural primary care clinics.
- A bundle of implementation strategies that include (1) academic-practice partnership, (2) development of implementation blueprint through distance-learning training modules, and (3) plan-do-study-act cycle for continuous refinement resulted in selection and adoption of evidence-based approaches for the two rural clinics with varying levels of implementation and modest improvements in CRCS outcomes.
- This bundle of strategies has potential to improve adoption, implementation, and sustainability of CRCS EBIs in rural primary care settings.

## Background

Colorectal cancer (CRC) is the fourth most common cancer in the USA [1]. In 2022, approximately 150,000 individuals are estimated to be diagnosed with CRC, and over 50,000 individuals will die from CRC in the U.S. CRC has the second highest cost of cancer in the USA. According to the projection of 2010–2020, total annual medical cost for CRC was $14.1 billion and average Medicare health care spending for patients with newly diagnosed CRC ranges from $40,000 to $80,000 depending on the stage [2, 3]. CRC is among the few preventable cancers through screening. The United States Preventive Services Task Force (USPSTF) rated CRC screening the highest-ranking (“A” grade) for a preventive care screening [4]. Anyone aged between 45 and 75 without symptoms or family history can prevent or detect cancer in earlier stages by performing visual examinations (e.g., colonoscopy) or taking high-sensitive stool tests (e.g., fecal immunochemical test). Yet, CRC screening (CRCS) rate in the USA is not optimal. According to 2020 Behavioral Risk Factor Surveillance System data, 69.7% of Americans aged 50–75 met the USPSTF recommendation [5], yet below than the 74.4% goal by Healthy People 2030 [6] and 80% goal by the National Colorectal Cancer Roundtable [7].

Rural communities experience much lower CRCS rates than urban counterparts. In 2013, two studies found rural-urban disparities among CRCS. Studies reported that rural patients were less likely to receive screening recommendation by physicians, and less likely to have completed screening or the stool-based test compared to their urban counterparts [8–11]. For example, a recent survey study conducted in rural Nebraska found that rural primary care patients were less likely to have CRCS compared to their urban counterparts (74.4% vs. 88.1%, *p* < 0.001) [10]. This rural-urban gap is exacerbated when combined with racial and ethnic factors (e.g., rural with higher proportion of Hispanic populations having lower CRCS rates) and is even larger in states with lower screening rates compared to those with higher screening rates [12].

A plethora of evidence-based intervention (EBI) strategies and programs to promote CRCS are available. The Community Guide recommends more than 15 intervention strategies to increase community demand (e.g., one-on-one education or client reminders), community access (e.g., reducing structural barriers by providing navigation services), or increase provider delivery (e.g., provider assessment and feedback) [13–16]. The National Cancer Institute also introduced 22 research-tested intervention programs (RTIPs) for promoting CRCS, and among those, eight targeted rural and low-income populations [17]. More recently, direct mailing strategies using stool-based CRCS tests were shown to be highly effective, combined with other strategies (e.g., education, navigation, or client reminders) [15, 18–21].

Despite these strong evidence and a wide range of selections, adopting and implementing CRCS EBIs is still challenging for rural health practitioners [22]. Some of the major challenges for rural systems include locating the most up to date evidence [8], determining the resources and system changes needed for implementation, and then selecting and initiating the EBIs that fit best to practice organizations’ context [23, 24]. This is not an inconsequential issue because uncertainty about “fit” can potentially lead to poor outcomes in the implementation and sustainability of EBIs. Uncertainty can also put heavy demands on rural health systems that are chronically overburdened and under-resourced, further increasing concerns about applying resources to strategies that ultimately do not fit within the local system.

To address these challenges in rural practices, the study introduced the co-production of implementation strategies to facilitate CRCS EBI uptake in the rural health systems. Co-production (also called, co-design) is “a collaboration between researchers and end users (rural health systems) from the onset, in question framing, research design and delivery, and influencing strategy, with implementation and broader dissemination strategies part of its design from gestations.” [25]. Co-production is often operationalized as a participatory approach that includes an ongoing, engaged clinical-academic partnership to facilitate the movement of EBIs from research to practice [26–28].

Participatory approaches also tend to bundle implementation strategies to help facilitate the translation of EBI to practice. Using Expert Recommendations for Implementing Change (ERIC) taxonomy, we bundled three implementation strategies: (1) developing an academic-clinical partnership, (2) creating a formal implementation blueprint through an online blueprinting process, and (3) using plan-do-study-act (PDSA) cycles to identify clinical partner interests and preferences on delivery methods, content refinement, and system change processes that improve CRCS EBI adoption implementation and sustainability [29]. In this study, we described the process of our co-production (participatory) approach including the development and delivery of the bundle of implementation strategies and tested the feasibility and acceptability of this bundled approach with the two rural clinics.

## Methods

### Study setting and participants

The study was conducted with two rural primary care clinics as a part of an accountable care organization (ACO) located in a rural county with a population of 34,914 (2019 estimates by US Census) with a Rural-Urban Commuting Area (RUCA) Codes of “4 = micropolitan area core with primary flow within an urban cluster of 10,000 to 49,999” or “5 = micropolitan high commuting with primary flow 30% or more to a large urban cluster” [30]. The participating ACO was consisted of a 116-bed regional referral center and six primary care clinics. All six clinics and a regional referral center are located in the same county. Through initial meetings, we identified that CRCS is one of the priority areas of the ACO since they have participated in a value-based payment program for commercially insured patients since 2018.

Among the six clinics, the two clinics showed interests in participating in the study. Clinic A provides essential primary care services to the community through six providers (three physicians, an Advance Practice Registered Nurse [APRN], and two Physician Assistants [PAs]) and additional 17 staff members. Clinic B is the largest primary care practice in the ACO network and provides services through 11 providers (six physicians, two APRNs, a PA, and two residents) with the support of 22 non-medical staff. Both clinics have participated in the co-production approach from July 2019 to March 2022 (see Additional file 1 for project milestone).

Three to five representatives from each clinic participated in the study. Following guidance from the literature on systems-based approaches [26], we recommended each clinic to have a team composed of at least one “decision-maker” (e.g., lead physician or manager) and one “doer” who carries out implementation plans (e.g., clinical care coordinators or frontline staff) and “supporter” who provide additional support to the team (e.g., data specialists, or other administrative staff). The ACO leadership team was also invited to, and engaged in, providing feedback on the process.

### Study design

This study applied the principles of participatory action research that uses a “reflection, data collection, and action that aims to improve health and reduce inequalities through involving the people who, in turn, take actions to improve their own health” [31]. In our study, we involved key partners (rural ACO clinic providers) in identifying problems (CRCS), reflecting on past and current approaches to promote CRCS, and taking action with a new approach (e.g., co-produced strategies).

### Intervention (bundled implementation strategies)

**Fig. 1:**
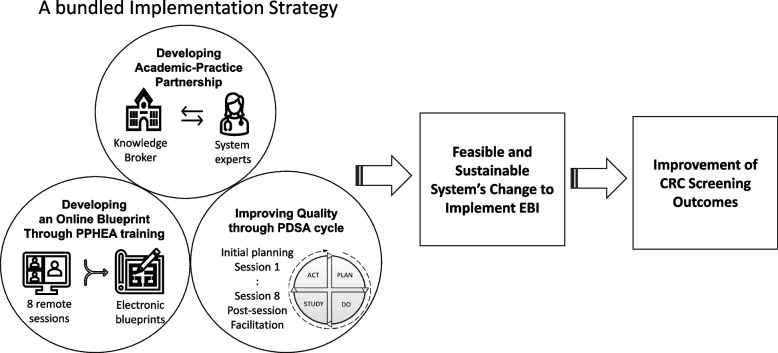
A bundled implementation strategy to facilitate evidence-based interventions to promote colorectal cancer screening

Our co-production approach used a bundle of the three implementation strategies (Fig. 1) to facilitate rural clinic partners to locate, select, and implement CRCS EBIs in their practices.

#### Academic-practice partnership

Following the approach by Estabrooks and his team [26], we integrated an academic-practice partnership through ongoing interactions with our clinical partners (ACO) on the process of problem prioritization, strategy selection, adaptation, trials, evaluation, and decision-making. The role of academic members in the partnership is to increase resources by engaging as “knowledge brokers” that can summarize existing EBIs, provide support for health systems to prioritize across available EBIs, and gather and report on system processes and outcomes that can inform adaptation and sustainability. The role of clinical members is to bring staff, organizational knowledge, experience, and culture together by engaging as “system experts.” The partnership enables locating and selecting the EBIs that best align with practice needs and capacities, and determining system changes necessary for implementation.

#### Developing a blueprint for EBI implementation

A blueprint is a formal implementation plan or protocol that includes the (1) aim/purpose of the implementation, (2) scope of the change, (3) timeframe and milestones, and (4) appropriate performance/process measures [29]. Once developed, the blueprint can be used and updated to guide the implementation effort over time. To facilitate the development of the blueprint, we adapted “Putting Public Health Evidence in Action (PPHEA)” curriculum developed by the Cancer Prevention and Control Research Network [32]. PPHEA curriculum provides eight publicly available, ready-to-use training sessions and tools to guide each step required to adapt, implement, and evaluate EBIs to promote various public health programs [33–35]. While these applications of the PPHEA show promise, initial applications used a relatively intensive face-to-face process that is unlikely to fit in busy, rural primary care practices. To further increase the usability of this approach, we developed an online blueprinting process that uses distance-learning modules to deliver the PPHEA training. The online module development team consisted of an academic team including an implementation scientist (PE), health services researcher (JK), distance-learning instructional designer (AM), and research assistant (AA) as well as rural health system experts from the two clinics.

#### Plan-Do-Study-Act (PDSA) cycle

We used the PDSA cycle as a key approach within our bundled implementation strategy [36]. In the planning stage, we conducted two qualitative focus groups (*n* = 8) to assess clinic representatives’ opinions regarding the most suitable methods to receive information on EBI characteristics and blueprints for implementation, systems-change strategies to facilitate implementation, and tools to identify intervention strategies that are both efficient and effective (Plan). Each focus group session took about an hour and conducted in person at each clinic’s conference room. Based on information from the planning session, we delivered a prototype distance-learning module of PPHEA training to clinic participants (Do). Upon completing each session (*n* = 8 sessions), participants provided feedback using surveys to assess perceptions of the bundled approach and potential adaptations for the next session (Study). Based on the feedback, modifications were made for the next modules (Act). After delivering all eight sessions, academic facilitators continued to hold monthly meetings with clinic participants to facilitate the implementation of the EBIs selected from the training (Additional File 1).

### Evaluation plan and measures

#### Participants’ reactions and perceptions

We adapted the post-training evaluation measures in the PPHEA training guide [34]. We administered an online survey asking participants’ reactions after completing each module regarding [1] overall satisfaction, (2) knowledge enhancement, (3) relevance to the job, (4) time investment, and (5) credibility of information. All items were rated on a 5-point Likert Scale of 1 = strongly disagree to 5 = strongly agree. After completing the first and last module, we evaluated participants’ perceptions of the bundled approach using Rogers’ Innovation Characteristics Measures [37] that included Relative Advantage (7 items), Compatibility (5 items), Simplicity (6 items), Trialability (3 items), and Observability (4 items). We adapted the questionnaires from the three existing studies [38–40]. See Additional File 2 for detailed survey instruments. Participants received a $10 gift card for completing each survey and an additional $20 gift card for completing all eight surveys.

#### Adoption, implementation, and outcomes of EBIs

Adoption was defined as “Yes” if any of the activities or plans of the selected EBIs were initiated at the clinic based on the monthly facilitation meeting notes recorded by a research team staff. Implementation was measured as the proportion of the activities or plans completed as compared to all the activities or plans developed in the formal implementation blueprint document. For example, if there are five activities or plans developed in the blueprint and three has completed, we recorded 60% implementation rates. CRCS outcome data were limitedly available at both clinics. Clinic A provided overall CRCS rates between fiscal year 2020 and fiscal year 2021 based on an annual performance report developed for commercially insured patients (about 50% of the entire clinic population). Clinic B provided number and proportions of CRCS eligible patients who completed the screening during the flu vaccination season (August to February of 2020 and 2021).

### Analysis

We used descriptive statistics, including Means, Standard Deviations, Frequencies, and Percentages, to analyze quantitative data. Qualitative data (initial focus group) were analyzed by inductive and deductive development and organization of thematic codes. The research team (JK and AA) developed a coding structure, which includes key conceptual domains and participant perspectives. Minor modifications were made iteratively until the model was saturated. Facilitation notes were carefully reviewed and summarized. Data were analyzed using SAS version 9.3 and NVivo qualitative analysis software (QSR NVivo 11).

## Results

### Development of the distance-learning modules (PPHEA training) tailored to CRCS

Through the initial focus group, we identified clinic participants’ interests and preferences on the content tailored to CRCS and delivery methods using synchronous (real-time video conferencing) and asynchronous (pre-recorded lecture video) distance learning technologies. Based on these initial preferences, we converted the original eight PPHEA sessions to pre-recorded, online video sessions followed by online discussion forums or live-streaming conference videos/calls facilitated by the academic team. Following the PPHEA training facilitator’s guide, we included all core contents in each training session and customized contexts/supplemental materials (e.g., handouts or tools) specific to CRCS EBIs. This resulted in the integration of 6 EBI strategies recommended by the CommunityGuide (small media, client reminder, one-on-one education, provider feedback and assessment, and provider reminder, reducing structural barriers) and three packaged programs introduced by Research Tested Intervention Programs (Flu-FIT/FOBT, Community Cancer Screening Program, and FIT & Colonoscopy Outreach) as well as the recent evidence of mailed stool-based approaches and multi-component strategies [18–21]. Original and modified PPHEA module contents are illustrated in the Additional File 3. We used a free online learning management system (LMS) called “Moodle” developed and maintained by the University of Nebraska Medical Center to upload module contents and communicate with learners via online discussion forums. For real-time video conferencing, we used “Zoom” or “Webex.”

### Delivery of distance-learning modules

The modules were delivered to the clinic participants on a monthly interval from October 2019 to August 2020, except the 2 months that were affected by the COVID-19 outbreak. The team composition grew relatively organically within each clinic and differed for the two clinics. Clinic A’s team consists of three primary care providers (two physicians and an APRN) and a nurse clinical manager from the ACO administrative team. Clinic B’s team included a physician, a clinical data coordinator, a nurse care coordinator, a referral/schedule coordinator, and a care manager. The two clinical teams also showed different learning styles. Clinic A used a “*group learning*” approach (viewed online lectures together at a reserved conference room followed by live streaming discussion). In contrast, clinic B used a “*hybrid learning*” approach (individuals viewed online lectures separately to cover material before a group meeting and video conference facilitated by the academic partners). After implementing the first module (defining EBI), clinic A provided constructive feedback regarding the video lecture presentation quality and content (e.g., too monotonous; less dynamic). Clinic A also requested to skip the session on community assessment and move directly to the module session that included the CRCS EBI examples. After receiving clinic’s feedback, we improved the video presentation quality and skipped the module 2 (community assessment). As a result, clinic A completed seven sessions, while clinic B completed all eight sessions as planned (see Table 1).

**Table 1:** Original and adapted plan for the module delivery

	Original plan	Adaptation
Team Composition	• Having an interdisciplinary team including decision-makers, implementers, and support staff	• Clinic A team included 3 providers (2 physicians and 1 APRN) and a nurse care manager hired by the ACO • Clinic B team included 1 lead physician, 1 clinical data coordinator, 1 nurse care coordinator, 1 referral/scheduling coordinator, and 1 care manager
Format	• 15–20 minutes video lectures through learning management system (LMS) followed by 30-minute live-streaming conference call/video	• Clinic A prefers to receive information (video links and surveys) via email rather than using web based LMS. • Clinic B adopted LMS.
Learning approach	• Participants are expected to watch video individually and come to live-streaming session for group discussion.	• Clinic A prefers to watch the video together followed by group discussion in a reserved conference room (**group learning approach**). • Clinic B prefers to watch lecture video individually followed by group discussion via live-streaming conference (**hybrid learning approach**)
Delivery	• Deliver all 8 sessions in a monthly interval (8 months). • Up to 3 weekly reminders to complete video lectures prior to live-streaming sessions	• Delivered sessions in a monthly interval except the two months affected by COVID-19 (10 months) • Clinic A: 7 sessions were delivered (Skipped session 2 after receiving feedback that they would like to minimize basic definition parts and jump right into the EBI examples). • Clinic B: All 8 sessions were delivered as planned.

### Participants’ reactions

Despite some negative feedback from clinic A for the first session, participants in both clinics reported overall high mean scores (most scores 4 points or higher on a scale of 1 = “strongly disagree” to 5 = “strongly agree”) on the five items of reactions: overall satisfaction with the session, knowledge enhancement on CRCS interventions, relevance to job, worth the time invested, and credibility of information (see Table 2).

**Table 2:** Participants’ reaction to the implementation strategy

	Mean (SD)								
	Module 1	Module 2	Module 3	Module 4	Module 5	Module 6	Module 7	Module 8	All
I was satisfied with this session overall									
Clinic A	3.9 (0.4)	-	4.4 (0.6)	4.2 (1.3)	4.2 (0.8)	-	4.7 (0.6)	4.3 (0.6)	4.2 (0.8)
Clinic B	4.8 (0.5)	4.2 (0.8)	4.0 (0.0)	4.0 (0.8)	4.3 (0.6)	4.7 (0.6)	4.0 (0.0)	4.7 (0.6)	4.4 (0.6)
This session enhanced my knowledge on planning CRC screening interventions									
Clinic A	3.9 (0.4)	-	4.4 (0.6)	3.8 (1.5)	4.0 (0.7)	-	4.7 (0.6)	4.3 (0.6)	4.1 (0.8)
Clinic B	4.0 (0.8)	4.0 (0.7)	4.0 (1.0)	4.3 (0.6)	4.3 (0.6)	4.3 (0.6)	4.0 (0.0)	5.0 (0.0)	4.2 (0.6)
This session provided content that is relevant to my daily job									
Clinic A	3.4 (0.8)	-	4.4 (0.6)	4.7 (0.5)	4.0 (1.0)	-	4.3 (0.6)	4.7 (0.6)	4.2 (0.8)
Clinic B	4.3 (1.0)	4.0 (0.7)	4.0 (0.0)	3.8 (0.5)	4.0 (0.0)	4.3 (0.6)	4.0 (1.0)	4.3 (0.6)	4.1 (0.6)
The gains that I have received from this session have been worth the time that I invested									
Clinic A	3.7 (0.5)	-	4.4 (0.6)	4.5 (1.2)	3.8 (0.8)	-	4.3 (0.6)	4.3 (0.6)	4.0 (1.0)
Clinic B	4.5 (0.6)	4.0 (0.7)	4.0 (0.0)	3.8 (0.5)	4.0 (0.0)	4.0 (0.0)	4.7 (0.6)	5.0 (0.0)	4.2 (0.5)
The session contained credible information about the topic									
Clinic A	3.9 (0.4)	-	4.7 (0.5)	4.4 (0.5)	4.4 (0.9)	-	4.3 (0.6)	4.7 (0.6)	4.4 (0.6)
Clinic B	4.5 (0.6)	4.0 (0.7)	4.0 (0.0)	4.0 (0.8)	4.3 (0.6)	4.3 (0.6)	4.7 (0.6)	5.0 (0.0)	4.3 (0.6)

Note: Survey data not available for Module 2 and 6 (Clinic A). We used Likert scale of 1 = ”strongly disagree” to 5 = ”strongly agree”

### Participants’ perception of innovation (i.e., bundled implementation strategy) characteristics

Both clinics reported improvements in their perceptions of the bundled implementation strategy after completing all the distance-learning sessions, although differences vary by characteristic domain and clinic. In clinic A, the largest improvements were shown in Relative Advantage (Diff = 1.43) followed by Trialability (Diff = 1.34). In clinic B, the largest improvement was seen in their perception of Compatibility of the bundled approach (Diff = 0.72) (see Table 3).

**Table 3:** Participants’ perceptions toward innovation characteristics (before and after)

	Clinic A			Clinic B		
	Baseline (*n=*7)	Follow-up (*n=*3)	Diff	Baseline (*n=*4)	Follow-up (*n=*3)	Diff
Relative advantage	3.00	4.43	1.43	3.86	4.52	0.66
Compatibility	4.00	4.40	0.40	3.95	4.67	0.72
Simplicity	3.19	4.50	1.31	4.25	4.56	0.31
Trialability	3.33	4.67	1.34	4.17	4.33	0.16
Observability	3.42	4.44	1.02	4.00	4.11	0.11

Note: We measured these items after the first session (baseline) and after the last session (follow-up). We took the average score of multiple items that are associated with each Innovation Characteristics domain. We used Likert scale of 1 = ”strongly disagree” to 5 = ”strongly agree”

### Adoption, implementation, and outcomes of the selected EBIs

After completing all the modules, both clinics developed a specific plan to implement CRCS EBIs (Table 4). Clinic A chose a combination of small media and client reminder intervention using mailed postcards informing patients regarding CRCS followed by telephone reminders. Clinic B developed an idea to adapt Flu-FIT/Flu-FOBT program, which uses an injection nurse to recommend CRCS for patients who visit the clinic for receiving the flu vaccine. Both clinics developed a formal implementation blueprint for the selected EBIs that included specific goals and activities, person responsible, resources, progress, and indicators of completion.

**Table 4:** Adoption, implementation, and outcomes of the selected EBIs

	Clinic A	Clinic B
EBIs selected	Small Media; Client Reminder (Source: CommunityGuide)	Flu-FIT/FOBT (Source: RTIPs)
EBI descriptions	Mailing a postcard to patients aged 50-75 who are due for CRCS to inform patients regarding CRCS followed by telephone reminders	Having injection nurses to recommend CRCS for patients who visit the clinic for receiving the flu vaccine (Sept – Feb)
Adaptation	Target patients covered by a large commercial plan (about 50% of clinic patients) Use the quarterly ‘gap’ report shared by the commercial plan to identify target population and track performances Sending 20 postcards at a time (rather than a mass-mailing) on a biweekly basis, and followed by phone reminder	-Target patients who physically visit clinic for a flu-shot and who are due for CRC screening -Recommend all options for CRCS test -Instead of mailing postcards/letters, use social media for advertising (facebook) -(COVID-19 specific) Train medical assistants for injection due to nurse shortage -(COVID-19 specific) Use curbside injection at the clinic parking lot
Activities planned and implementation status	1. Prepare postcards and pamphlets with tailored messages and design for target group (*implemented*) 2. Mail out 1st batch of postcards (*n=*20) and complete follow up calls (implemented with 6 month delay) 3. Create a tracking log, update the mailing/phone call status, and share with the team (*partially implemented*) 4. Assess pilot runs and plan for next cycle (*implemented*) 5. Mail out 2nd batch of postcards and complete follow-up calls (*not implemented*) 6. Mail out 3nd batch of postcards and complete follow-up calls (*not implemented*) Note: 3.5/6 = 58% implemented (implementation delayed about 6 month due to loss of a lead physician and staff turnover. A support staff not in the training took over the implementation on July 2021).	1. Prepare resources (flu vaccine shipment, CRCS handouts, signs for curbside visit, FIT/FOBT kit) (*implemented*) 2. Meet and communicate injection nurses, referral coordinator, and frontline staff and confirm the workflow (*implemented*) 3. Run pilot test (Oct, 2020 – Feb, 2021) (*implemented*) 4. Assess pilot runs and plan for next cycle (*implemented*) 5. Run second implementation cycle with revised plan (Oct, 2021 – Feb 2022) (*implemented*) 6. Assess 2nd cycle and develop plans for next year (*implemented*) Note: 6/6 = 100% implemented (implementation went smoothly according to initial plans with a few adaptations).
CRCS uptake outcomes	Between July 2021 and January 2022, 34 postcards were sent and 24 follow up calls completed. Placed 1 colonoscopy referral and 1 FIT-DNA ordered; Between FY20 and FY21, there was an increase of CRCS from 71% to 77% (gap report).	**Year 1 (8/1/20 – 2/15/21)**: 977 came for flu shot; of these, 163 were due for CRCS. Of 163, 29 (17.8%) completed CRCS within the 6 months. **Year 2 (8/1/21 – 2/15/22)**: 1175 came for flu shot; Of these, 214 were due for CRCS. Of 214, 38 (17.7%) completed CRCS within the 6 months.

Upon the completion of the online blueprinting process through the distance-learning PPHEA modules, the academic team continued to meet with each clinic team monthly to facilitate the adoption and implementation of the selected EBI (October 2020 through March 2022). As shown in the Table 4, both clinics adopted the EBIs, with varying degrees of implementation. Due to the surge of patient care needs during the COVID-19 pandemic and staff turnover, clinic A delayed the implementation about 6 months, and implemented only 58% of the intervention activities planned. Between July 2021 and January 2022, 34 postcards (about 35% of the total number of mailings initially planned) were sent and 24 follow-up calls were completed. This resulted in one colonoscopy referral and one FIT-DNA ordered. According to the gap report, between the FY20 and FY21, there was an increase of CRCS from 71 to 77%; however, it is not clear whether this increase was solely accounted for the EBIs that were implemented. Clinic B was able to achieve 100% implementation for the Flu-CRC program. During the pilot implementation trials in the year 1 (August 2020–February 2021), 977 patients visited the clinic B for a flu shot. Of these, 163 were due for CRCS were recommended by injection nurses to schedule or order screening tests. This resulted in 29 patients completed CRCS within the six months. In the year 2 (August 2021–February 2022), 1175 patients came for a flu shot, and 214 were due for CRCS. Of these, 38 completed CRCS within the 6 months.

## Discussion

The selection and implementation of CRCS EBIs in rural primary care clinics are critical given the geographic disparities in cancer screening and outcomes. Equally important is understanding how these clinics perceive co-production of implementation strategies intended to facilitate the uptake of CRCS EBIs. In this study, we began our approach by building an academic-practice partnership in the process of problem prioritization (CRCS), strategy selection, adaptation, and implementation. We developed implementation blueprints by providing training and education using the adapted PPHEA modules specifically designed for, and delivered to rural primary care practitioners. We also used a rapid improvement cycle (PDSA) to make iterative changes to the implementation strategy based on clinic staff feedback. Our approach was perceived positively to clinic participants and resulted in an adoption of EBIs in each clinic with varying levels of implementation. Our project provides preliminary information about the potential of the bundled implementation strategy as feasible and acceptable in rural primary care practices.

While this is preliminary and pilot from two rural clinics, our findings align with research that suggests the likely success of implementation strategies that bundle activities such as academic-practice partnerships, implementation blueprint, and quality improvement strategies with regular feedback with iterative adaptations [41, 42]. Adams and colleagues (2018) conducted a survey study of key informants at federally qualified health centers (FQHCs) in eight states to examine which EBIs to promote CRCS were used, and which implementation strategies were employed. They found that FQHCs used multiple implementation strategies (an average of 10, range 2–19) as “bundles” depending on different implementation stages. A few examples of such strategies include identifying barriers to implementing evidence-based approaches, consistently monitoring the implementation process and modifying as appropriate, distributing CRC guidelines and EBI materials to providers, and developing a formal implementation blueprint. Particularly, the Adams et al. work highlighted the potential gains of training health system staff on process of developing and executing implementation strategies (e.g., developing a formal implementation blueprint or conducting group educational meetings for providers) consistent with the previous studies underscoring the need for more support and guidance for EBI implementation [43, 44]. Our findings suggest a feasible and acceptable way to work with these health systems, especially in the rural areas, to provide guidance and resources for selecting and implementing EBIs to promote CRCS.

It is worth noting that our co-production approach was well received by our rural primary care practitioners given that most participants reporting positive satisfaction and relative advantage of the bundled implementation strategy approach. Specifically, participants from both clinics reported significant improvements in relative advantage and compatibility, which indicates a relative benefit of our bundled approach compared to other implementation strategies and a better fit with the clinics’ needs and capacity. While previous studies focus on the volume of system-level implementation strategies (e.g., more strategies correlate with higher screening rates) [41, 42], our findings add to the previous studies by highlighting that the number of strategies may be less important than having system strategies that align well with the local context. However, the degree to which this is related to successful implementation of EBIs and CRCS outcomes will need to be tested in a larger trial.

Interestingly, the two clinic teams showed varying levels of implementation. Besides the struggle that the clinic A faced due to the loss of a lead physician in the midst of pandemic, clinic A did not engage any support staff or “doers” in the module training process, which could be the reason for delayed and partial implementation. This goes back to the literature highlighting the importance of involving interdisciplinary staff (practice manager, frontline office delivery staff, data person) in the implementation team composition depending on the scope and complexity of the project [26]. Daly et al.’s study also underscored the importance of engaging office support staff encouraging CRCS as one of the key system-level strategies used in community health centers serving medically vulnerable patients. Additionally, some of our rural primary care participants wanted less education on community assessment and definitions and quicker access to modules that provided example CRCS EBIs. An alternative approach may be to combine modules (e.g., module 1: defining EBIs and module 2: community assessment) or to reduce the modules that are not the core components (e.g., module 8: communication). Future studies may consider the engagement of program delivery staff more fully in the development of blueprint processes [42] and examine the pace and content of the module delivery based on local needs and context while minimizing the changes in the core component of the training.

While only a pilot, the process supported both clinics in identifying and initiating implementation of evidence-based approaches to increase CRC screening. Both clinics selected EBIs that had been included as examples, underscoring the need to do preliminary work to ensure that a *range of examples* are provided to fit differential resources across rural clinics. Interestingly, our rural clinic partners prefer EBIs that include colonoscopy as a major test option and stool-based tests as alternatives, rather than selecting stool-based test as a single main intervention (e.g., direct mailing of FIT). We found the similar preferences in another rural-focused study reporting that rural clinics are more likely to prefer the use of colonoscopy alone or prefer to use both colonoscopy and stool tests [10, 45]. This may be due to the rural practitioners’ uncertainties around the effectiveness of the stool-based tests, or concerns about out-of-pocket cost for “diagnostic” colonoscopy when performed following a positive stool-based test, which is more expensive than screening colonoscopy. Future programs may need to include up-to-date scientific evidence on effectiveness of the stool-based tests as well as more accurate and transparent cost implications for each screening test modalities.

Of course, as a pilot project, there are several limitations. These include a small sample of clinics and staff from each clinic, and both clinics also came from the same region, which limits generalizability. We did not include patient/community representatives in our co-production process, which may lack their perspectives on the best fitting CRCS EBIs for the community’s needs. Finally, our quantitative data is limited to descriptive considerations rather than inferential statistics due to the small sample size. Nevertheless, our qualitative data indicated that this participatory approach fits well with the clinical resources, time, and interest. Future work will include testing the newly developed modules in a broader range of rural clinics to determine the utility of this co-production of the bundled implementation strategies in supporting EBI selection, adoption, implementation, and sustainability to promote CRCS.

## Conclusions

Little is known about the co-production of implementation strategies for evidence-based CRCS in rural primary care clinics. We used a bundle of strategies (developing an academic-clinical partnership, forming an implementation blueprint, implementing quality improvement strategies to provide regular feedback and iterative adaptations) to help rural clinics identify the best fitting EBI to their practice context. We developed eight distance-learning modules to build an online blueprint for CRCS EBI selection and implementation combined with monthly live-streaming conferences to allow for CRCS tailoring. After completing all the modules, participants in two rural clinics reported positive reactions toward the bundled approach. Both clinics reported improvements in how they perceived the characteristics of the bundled approach to tailor selected CRCS EBIs. Through the process, both clinics developed and adopted the EBIs with varying degrees of implementation and modest increase of CRCS outcomes. Our preliminary data showed that our bundle of implementation strategies were feasible and acceptable in rural primary care practices with modifications for the local context to facilitate the use of evidence-based approaches to improve CRCS.

## Supplementary Information

**Additional file 1..** Project Milestone

**Additional file 2..** Survey Instruments

**Additional file 3..** Original and Modified PPHEA Modules with video links

**Additional file 4..** TIDIeR checklist

## Data Availability

The datasets used and/or analyzed during the current study are available from the corresponding author on reasonable request.

## Abbreviations

ACO: Accountable care organization
APRN: Advance Practice Registered Nurse
CRC: Colorectal cancer
CRCS: Colorectal cancer screening
EBI: Evidence-based intervention
PA: Physician assistant
PCP: Primary care provider
PDSA: Plan-Do-Study-Act
PPHEA: Putting Public Health Evidence in Action
USPSTF: United States Preventive Services Task Force
