# Supplementary material for: Developing a co-production strategy to facilitate the adoption and implementation of evidence-based colorectal cancer screening interventions for rural health systems: a pilot study
Source: Implement Sci Commun. 2022 Dec 13;3:131. doi: 10.1186/s43058-022-00375-2 (PMC9745718; doi:10.1186/s43058-022-00375-2)
Supplement: Supplementary file 1 — Additional file 1.. Project Milestone [file 43058_2022_375_MOESM1_ESM.docx]

**Supplementary Table 1. Project Milestone**

| Activities | 2019 | | 2020 | | | | 2021 | | | | 2022 |
| --- | --- | --- | --- | --- | --- | --- | --- | --- | --- | --- | --- |
|  | Q3 | Q4 | Q1 | Q2 | Q3 | Q4 | Q1 | Q2 | Q3 | Q4 | Q1 |
| Initial focus group | X |  |  |  |  |  |  |  |  |  |  |
| PPHEA module development & delivery |  |  |  |  |  |  |  |  |  |  |  |
| Clinic A |  | X | X | X | X |  |  |  |  |  |  |
| Clinic B |  | X | X | X | X |  |  |  |  |  |  |
| Post-module survey (reaction) |  | X | X | X | X |  |  |  |  |  |  |
| Post-module survey (perception of innovation) |  | X |  |  | X |  |  |  |  |  |  |
| Post-PPHEA facilitation monthly meeting |  |  |  |  |  |  |  |  |  |  |  |
| Clinic A |  |  |  |  |  |  |  | X | X | X | X |
| Clinic B |  |  |  |  |  | X | X | X | X | X | X |
| EBI implementation |  |  |  |  |  |  |  |  |  |  |  |
| Clinic A (Small media + Reminder) |  |  |  |  |  |  |  | X | X | X | X |
| Clinic B (Flu-CRC) |  |  |  |  |  | X | X |  |  | X | X |
| Outcome data collection |  |  |  |  |  |  |  | X |  |  | X |
