# Supplementary material for: Developing a co-production strategy to facilitate the adoption and implementation of evidence-based colorectal cancer screening interventions for rural health systems: a pilot study
Source: Implement Sci Commun. 2022 Dec 13;3:131. doi: 10.1186/s43058-022-00375-2 (PMC9745718; doi:10.1186/s43058-022-00375-2)
Supplement: Supplementary file 2 — Additional file 2.. Survey Instruments [file 43058_2022_375_MOESM2_ESM.docx]

**Additional File 2. Survey Instruments**

**Post-Training Evaluation**

**Section 1. Reaction**

1. How much do you agree or disagree with the following statements?

|  | Strongly Disagree | Disagree | Neither | Agree | Strongly Agree | Don’t know |
| --- | --- | --- | --- | --- | --- | --- |
| 1. I was satisfied with this session overall | 1 | 2 | 3 | 4 | 5 | 99 |
| 1. This session enhanced my knowledge on developing a communication plan for evidence-based interventions for CRC screening | 1 | 2 | 3 | 4 | 5 | 99 |
| 1. This session provided content that is relevant to my daily job | 1 | 2 | 3 | 4 | 5 | 99 |
| 1. The gains that I have received from this session have been worth the time that I invested | 1 | 2 | 3 | 4 | 5 | 99 |
| 1. This session contained credible information about the topic | 1 | 2 | 3 | 4 | 5 | 99 |

2. For your experience level, was the session (check one):

 Too basic  About right  Too advanced

3. How would you describe the balance between lecture and interaction? (check one):

 Too much lecture  About right  Too much interaction

4. What did you like most about this session?

|  |
| --- |

5. Which part of this session will be helpful in developing a communication plan for the most suitable/ feasible CRC intervention in your practice?

|  |
| --- |

6. How can this training be improved?

|  |
| --- |

7. How often should we re-visit the training? (ask only after the last session)

- Every 6 months  Annually  Every 2 years  Others

**Section II. Innovation Characteristics**

8. Now, we would like to ask your perception about various characteristics of innovation.

**Innovation: the distance-learning training that helps healthcare personnel and systems select and implement evidence-based CRC screening interventions.**

How much do you agree or disagree with the following statements **about the training approach**?

| Relative Advantage:  Using the training approach… | Strongly Disagree | Somewhat Disagree | Neither | Agree | Strongly Agree | Don’t know |
| --- | --- | --- | --- | --- | --- | --- |
| 1. enables me to accomplish tasks related to CRC screening program more quickly | 1 | 2 | 3 | 4 | 5 | 99 |
| 1. improves the quality of work I do related to CRC screening | 1 | 2 | 3 | 4 | 5 | 99 |
| 1. makes it easier to do my job related to CRC screening | 1 | 2 | 3 | 4 | 5 | 99 |
| 1. enhances my effectiveness on the job related to CRC screening | 1 | 2 | 3 | 4 | 5 | 99 |
| 1. increases my productivity related to CRC screening | 1 | 2 | 3 | 4 | 5 | 99 |
| 1. is better than our current approach of selecting and implementing CRC screening interventions | 1 | 2 | 3 | 4 | 5 | 99 |
| 1. helped me learn about evidence-based CRC screening interventions more quickly and easily | 1 | 2 | 3 | 4 | 5 | 99 |

| Compatibility:  Using the training approach… | Strongly Disagree | Disagree | Neither | Agree | Strongly Agree | Don’t know |
| --- | --- | --- | --- | --- | --- | --- |
| 1. is compatible with all aspects of my work | 1 | 2 | 3 | 4 | 5 | 99 |
| 1. fits well with the way I like to work | 1 | 2 | 3 | 4 | 5 | 99 |
| 1. fits into my working style | 1 | 2 | 3 | 4 | 5 | 99 |
| 1. fits well with the systems of care delivery in my organization | 1 | 2 | 3 | 4 | 5 | 99 |
| 1. fits well with the existing values and beliefs of our organization | 1 | 2 | 3 | 4 | 5 | 99 |

| Complexity (Simplicity):  Using the training approach… | Strongly Disagree | Disagree | Neither | Agree | Strongly Agree | Don’t know |
| --- | --- | --- | --- | --- | --- | --- |
| 1. was clear and understandable | 1 | 2 | 3 | 4 | 5 | 99 |
| 1. made it easy to learn what I wanted to learn | 1 | 2 | 3 | 4 | 5 | 99 |
| 1. made the curriculum easy to follow | 1 | 2 | 3 | 4 | 5 | 99 |
| 1. made learning to make changes easy for me | 1 | 2 | 3 | 4 | 5 | 99 |
| 1. was not difficult to access on my computer | 1 | 2 | 3 | 4 | 5 | 99 |
| 1. reduced the difficulty someone might have doing the training activities. | 1 | 2 | 3 | 4 | 5 | 99 |

| Trialability | Strongly Disagree | Disagree | Neither | Agree | Strongly Agree | Don’t know |
| --- | --- | --- | --- | --- | --- | --- |
| 1. Before deciding to take the full approach across my organization, I was able to properly try it out in a small scale | 1 | 2 | 3 | 4 | 5 | 99 |
| 1. I was permitted to use the training approach on a trial basis long enough to see what it could do | 1 | 2 | 3 | 4 | 5 | 99 |
| 1. It didn’t cost much to try the training approach even if I don’t like it | 1 | 2 | 3 | 4 | 5 | 99 |

| Observability | Strongly Disagree | Disagree | Neither | Agree | Strongly Agree | Don’t know |
| --- | --- | --- | --- | --- | --- | --- |
| 1. I would have no difficulty telling others about the benefit of using it | 1 | 2 | 3 | 4 | 5 | 99 |
| 1. I believe I could communicate to others the consequences of using it | 1 | 2 | 3 | 4 | 5 | 99 |
| 1. Produces the results that are easy to see | 1 | 2 | 3 | 4 | 5 | 99 |
| 1. I would have difficulty explaining why using it may or may not be beneficial |  |  |  |  |  |  |

9. What is your email address: **___________________________________________**

**We ask this question to track your participation and provide you an incentive ($10 gift card).*

*This information will be kept confidential.*

**Thank you!**
