# Supplementary material for: Developing a co-production strategy to facilitate the adoption and implementation of evidence-based colorectal cancer screening interventions for rural health systems: a pilot study
Source: Implement Sci Commun. 2022 Dec 13;3:131. doi: 10.1186/s43058-022-00375-2 (PMC9745718; doi:10.1186/s43058-022-00375-2)
Supplement: Supplementary file 3 — Additional file 3.. Original and Modified PPHEA Modules with video links [file 43058_2022_375_MOESM3_ESM.docx]

**Additional File 3. Adaptation of Putting Public Health Evidence in Action Curriculum with Video Links**

|  | Original Contents | Adaptation |
| --- | --- | --- |
| Session 1: Defining evidence | - 25 minutes of interactive lecture with PPT slides | - Three, 6-minute video clips:   <https://youtu.be/uORPbERH6t4>  <https://youtu.be/FcCutsfFjuc>  <https://youtu.be/KdxTv3Uoh3A>   - Two online forums - 30-minute, live streaming conference video/call |
| Session 2: Conducting community assessments | - 40 minutes of interactive lecture with PPT slides and activity (community assessment worksheet) | - Three, 6-9 minute video clips:   <https://youtu.be/BVc3i9IuQ60>  <https://youtu.be/QmDHEYwbyEA>  <https://youtu.be/SOWtLfoit78>   - Modify handouts specific to CRCS (community assessment template; problem logic map; list of resources; goals and objectives worksheet) - An online forum - 30-minute, live streaming conference video/call |
| Session 3: Finding evidence | - 60 minutes of interactive lecture with PPS slides - activity (finding evidence scavenger hunt) - Handouts (resource list, finding scavenger hunt answer key) | - Two 8–10-minute video clips:   <https://youtu.be/KNd4KcfcoLQ>  <https://youtu.be/Es_XUF-YV6Y>   - Introduce EBIs specific to CRCS from CommunityGuide and RTIPS:   - Small media, client reminder, one-on-one education   - Provider feedback and assessment, provider reminder   - Reducing structural barriers (e.g., navigation, stool-based approach)   - Multi-component approach   - Flu-FIT/FOBT, Community Cancer Screening Program, FIT & Colonoscopy Outreach - Two online forums - 30-minute, live streaming conference video/call |
| Session 4: Selecting an EBI with the best fit | - 30-45 minutes of interactive lecture with PPT slides and activities (comparison tool activity with CRC and Nutrition; CRC EBI Description 1 and 2; Nutrition EBI Description 1 and 2) - Handout: EBI comparison Tool - blank | - Two 8–10-minute video clips:   <https://youtu.be/Mn63q3jh14s>  <https://youtu.be/JevtaxUwwdc>   - Modifying EBI comparison tool to understand which EBI (Patient navigator/ Injection Nurse) is best fit for the individual clinical practice based on priorities (health goals and objectives, delivery methods, organization, and community resources) and target population. - An online forum - 30-minute, live streaming conference video/call |
| Session 5: Adapting an EBI to fit your community | - 30-45 minutes of interactive lecture with PPT slides - Handouts: Adaptation guidance tool | - Two 10-minute video clips:   <https://youtu.be/8u33KOsffpQ>  <https://youtu.be/1TIRb5N10Uo>   - Identifying best fit EBI for the practice. Modifying adaptation guidance tool to define the changes that can/can’t be made to adapt the EBI in practice for better fit or compatibility with the target population (green/yellow/red things) - Two online forums - 30-minute, live streaming conference video/call |
| Session 6: Implementing an EBI | - 60 minutes of interactive lecture with PPT slides and activity (PDSA cycle activity) - Handouts: Organizational readiness checklist - PDSA template - Project charter template, workplan template, example body and soul work plan | - Two 10-12 minutes video clips:   <https://youtu.be/aYNZ_nZHTtM>  <https://youtu.be/SkzN7MHqdeY>   - Modifying implementation team charter to define aims and objectives for implementing EBI’s best fir for practice (Flu-FIT) including project scope, responsibilities, and stakeholders. - An online forum - 30-minute, live streaming conference video/call |
| Session 7: Planning for evaluation | - 35 minutes of interactive lecture with PPT slides and activity (planning for evaluation activity; evaluation plan template activity) - Handouts: evaluation designs, evaluation resource list; logic model example FluFIT; FluFIT description, Body and Soul description | - Two 10–15-minute video clips:   <https://youtu.be/wMSNUL_Og3g>  <https://youtu.be/07STO0UsfsQ>   - Understanding and modifying evaluation plan to define the performance metrics-, short- and long-term outcome evaluation, and staff responsible for implementation. - 30-minute, live streaming conference video/call |
| Session 8: Creating a communication plan for EBI | - 35 minutes of interactive lecture with PPT slides and activity (safe routes to school example) - Handouts: communication plan template; communication plan (blank) | - One 14-minute video clip:   <https://youtu.be/7u7RiieYPN0>   - Modifying and developing a communication plan for the EBI (Flu-FIT) to define goals, objectives, audiences, marketing and communication channels, and timelines. - An online forum - 30-minute, live streaming conference video/call |
