# Supplementary material for: Developing a co-production strategy to facilitate the adoption and implementation of evidence-based colorectal cancer screening interventions for rural health systems: a pilot study
Source: Implement Sci Commun. 2022 Dec 13;3:131. doi: 10.1186/s43058-022-00375-2 (PMC9745718; doi:10.1186/s43058-022-00375-2)
Supplement: Supplementary file 4 — Additional file 4.. TIDIeR checklist [file 43058_2022_375_MOESM4_ESM.docx]

**
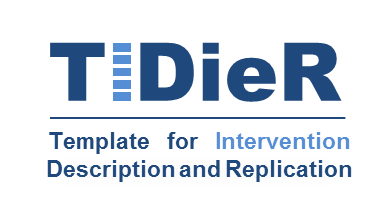
The TIDieR (Template for Intervention Description and Replication) Checklist*:**

Information to include when describing an intervention and the location of the information

| Item number | Item | Where located ** | |
| --- | --- | --- | --- |
|  |  | Primary paper  (page or appendix  number) | Other ^†^ (details) |
|  | **BRIEF NAME** |  |  |
| 1. | Provide the name or a phrase that describes the intervention. | p.8 (line 22) – p.10 (line 20) | A bundled implementation strategy (academic-practice partnership + development of implementation blueprint + plan-do-study-act cycle) |
|  | **WHY** |  |  |
| 2. | Describe any rationale, theory, or goal of the elements essential to the intervention. | p.6 (line 10) – p.8 (line 9) | The co-production of bundled implementation strategies help facilitate the translation of EBI to rural practice to promote colorectal cancer screening |
|  | **WHAT** |  |  |
| 3. | Materials: Describe any physical or informational materials used in the intervention, including those provided to participants or used in intervention delivery or in training of intervention providers. Provide information on where the materials can be accessed (e.g. online appendix, URL). | p. 9 (line 16) – p.10 (line 7); additional table 1. | The project adapted “Putting Public Health Evidence in Action (PPHEA)” to develop a blueprint and guide the implementation efforts over time. Additional file 1 included the original and modified modules of PPHEA curriculum. |
| 4. | Procedures: Describe each of the procedures, activities, and/or processes used in the intervention, including any enabling or support activities. | p.12 (line 8) – p.13 (line 20)  p.14 (line15) – p.15 (line 20) | See development of the PPHEA modules and delivery procedures in the results section.  See implementation and facilitation processes in the results section. |
|  | **WHO PROVIDED** |  |  |
| 5. | For each category of intervention provider (e.g. psychologist, nursing assistant), describe their expertise, background and any specific training given. | p.10 (line 4-7) | The online module development team consisted of an academic team including an implementation scientist (PE), health services researcher (JK), distance-learning instructional designer (AM), and research assistant (AA) as well as rural health system experts from the two clinics. |
|  | **HOW** |  |  |
| 6. | Describe the modes of delivery (e.g. face-to-face or by some other mechanism, such as internet or telephone) of the intervention and whether it was provided individually or in a group. | p.13 (line 4-21); See Table 1 | Module was delivered via online video followed by live conference, or online forum through the learning management software (Moodle system) and Zoom/Webex. |
|  | **WHERE** |  |  |
| 7. | Describe the type(s) of location(s) where the intervention occurred, including any necessary infrastructure or relevant features. | p.7 (line 15-22) – p.8 (line 1-15)  p.27-28 | See study setting in the methods section |
|  | **WHEN and HOW MUCH** |  |  |
| 8. | Describe the number of times the intervention was delivered and over what period of time including the number of sessions, their schedule, and their duration, intensity or dose. | p.13 (line 5-7) | The modules were delivered to the clinic participants on a monthly interval from October 2019 to August 2020, except the two months that were affected by the COVID-19 outbreak. |
|  | **TAILORING** |  |  |
| 9. | If the intervention was planned to be personalised, titrated or adapted, then describe what, why, when, and how. | p. 12 (line 9-22) | The PPHEA modules were adapted to rural practitioners’ needs and interests and tailored specific to colorectal cancer screening. |
|  | **MODIFICATIONS** |  |  |
| 10.^ǂ^ | If the intervention was modified during the course of the study, describe the changes (what, why, when, and how). | p.13 (line 4-22) | See Table 1 for the original and modified plan of delivery |
|  | **HOW WELL** |  |  |
| 11. | Planned: If intervention adherence or fidelity was assessed, describe how and by whom, and if any strategies were used to maintain or improve fidelity, describe them. | p.14-15; Table 4 | See Table 4 for more details about implementation fidelity assessed based on monthly facilitation notes by the research staff (facilitator) |
| 12.^ǂ^ | Actual: If intervention adherence or fidelity was assessed, describe the extent to which the intervention was delivered as planned. | p.14-15; Table 4 | See Table 4 for more details about implementation fidelity assessed based on monthly facilitation notes by the research staff (facilitator) |

** **Authors** - use N/A if an item is not applicable for the intervention being described. **Reviewers** – use ‘?’ if information about the element is not reported/not sufficiently reported.

† If the information is not provided in the primary paper, give details of where this information is available. This may include locations such as a published protocol or other published papers (provide citation details) or a website (provide the URL).

ǂ If completing the TIDieR checklist for a protocol, these items are not relevant to the protocol and cannot be described until the study is complete.

* We strongly recommend using this checklist in conjunction with the TIDieR guide (see *BMJ* 2014;348:g1687) which contains an explanation and elaboration for each item.

* The focus of TIDieR is on reporting details of the intervention elements (and where relevant, comparison elements) of a study. Other elements and methodological features of studies are covered by other reporting statements and checklists and have not been duplicated as part of the TIDieR checklist. When a **randomised trial** is being reported, the TIDieR checklist should be used in conjunction with the CONSORT statement (see [www.consort-statement.org](http://www.consort-statement.org)) as an extension of **Item 5 of the CONSORT 2010 Statement.** When a **clinical trial** **protocol** is being reported, the TIDieR checklist should be used in conjunction with the SPIRIT statement as an extension of **Item 11 of the SPIRIT 2013 Statement** (see [www.spirit-statement.org](http://www.spirit-statement.org)). For alternate study designs, TIDieR can be used in conjunction with the appropriate checklist for that study design (see [www.equator-network.org](http://www.equator-network.org)).
